# Global health post-2015: the case for universal health equity

**DOI:** 10.3402/gha.v6i0.19661

**Published:** 2013-04-03

**Authors:** Lucia D'Ambruoso

**Affiliations:** Umeå Centre for Global Health Research, Epidemiology and Global Health, Department of Public Health and Clinical Medicine, Umeå University, Umeå, Sweden

**Keywords:** Millennium Development Goals, post-2015 development, sustainable development, global health, equity

## Abstract

Set in 2000, with a completion date of 2015, the deadline for the Millennium Development Goals is approaching, at which time a new global development infrastructure will become operational. Unsurprisingly, the discussions on goals, topics, priorities and monitoring and evaluation are gaining momentum. But this *is* a critical juncture. Over a decade of development programming offers a unique opportunity to reflect on its structure, function and purpose in a contemporary global context. This article examines the topic from an analytical health perspective and identifies *universal health equity* as an operational and analytical priority to encourage attention to the root causes of unnecessary and unfair illness and disease from the perspectives of those for whom the issues have most direct relevance.

## Looking back: reflecting on the MDGs

**Fig. 1:**
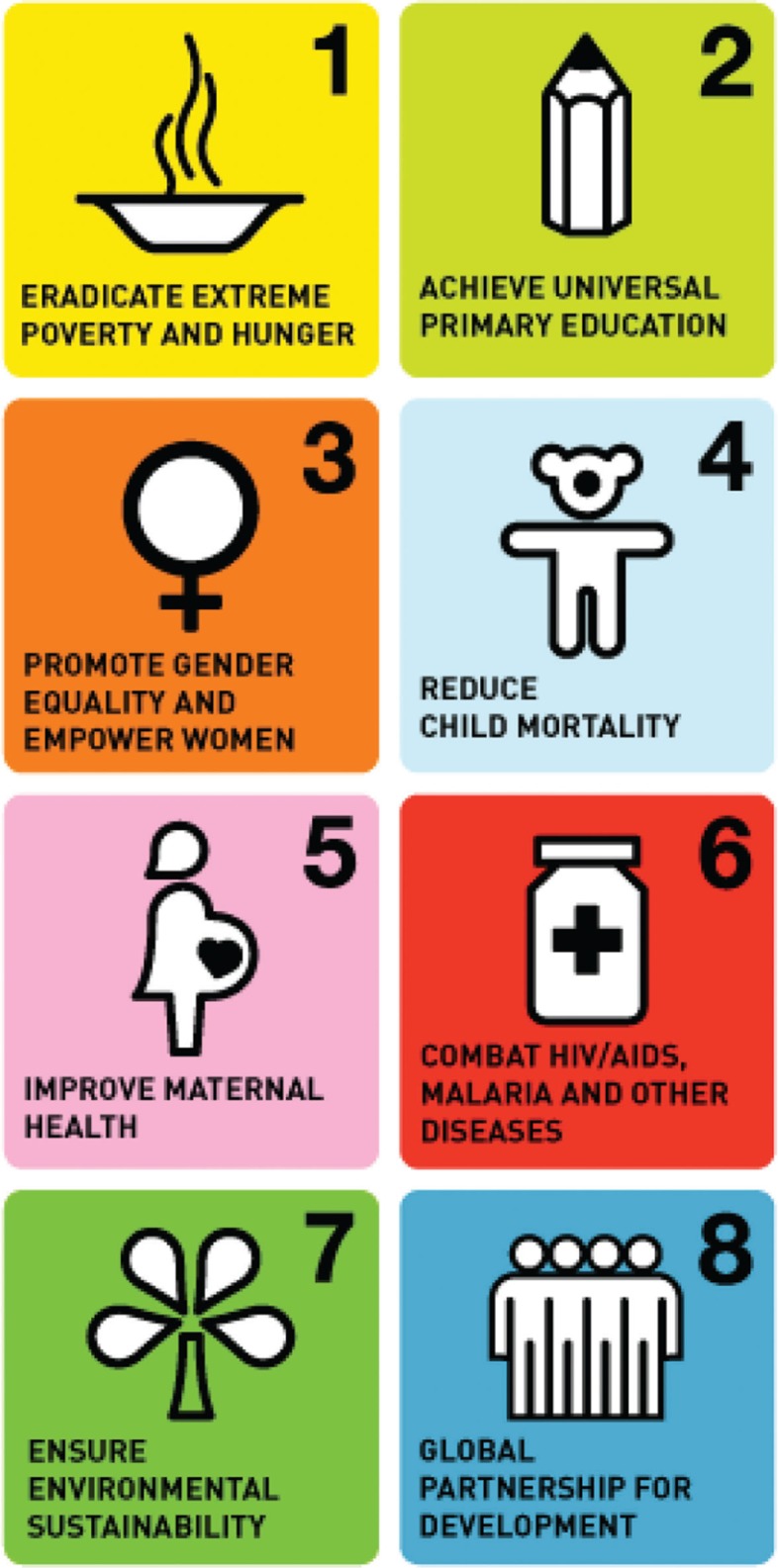
The Millennium Development Goals [reproduced from (1)].

The MDGs are a series of eight global goals that aim to improve education and health and reduce poverty (Fig. 1). The goals were derived from the Millennium Declaration, adopted by 189 UN member states in 2000, and later supplemented with targets and indicators to monitor progress (1). In little over a decade, extraordinary achievements have been attributed to the MDGs. These include the reduction of extreme poverty by 50%, improved conditions for 200 million people in slum areas, girls’ primary education enrolment equalling that of boys, significant reductions in rates of maternal and child mortality, tuberculosis and malaria, and over 6 million people with access to treatment for HIV/AIDS (2, 3).

It is important to understand what has enabled these achievements. In a recent review, Waage et al. reflect on the goals in terms of their design, political and historical contexts, implementation, and M&E (4). According to these authors, the main success was the extent of high-level political consensus that enabled a global campaign of development programming on an unprecedented scale. The ‘brand-value’ of the MDGs and the benefits of the holistic yet functional definition of poverty provided in the goals has also been identified as a feature that facilitated communication and enabled political buy-in (5).

Waage and colleagues also identify four main failures and missed opportunities characterising the MDGs. The first relates to fragmentation. In the health field alone: ‘little communication occurs between the communities of non-communicable disease, MDGs and the social determinants of health’ (6 p. 2194). The eight goals reflect this lack of inter-connectivity, prioritising topics over synergies, essentially promoting silos working. These gaps widened during implementation. At country level, numerous short-term, topic-specific, donor-driven projects implemented according to various timelines and operational and evaluatory frameworks gave rise to poor coordination, confusion, and unpredictable funding flows (7). More broadly, and particularly where there is consequential withdrawal of public service funding, the receipt of Official Development Assistance (ODA) can effectively disable states from the provision of effective coverage (8). Combined, these issues led to significant operational efficiency losses.

Waage et al. also describe limitations in the targets and indicators to capture the essence of the goals and, by extension, evidence of progress towards them. Illness and disease in resource poor settings are complex, biosocial phenomena that require multidimensional concepts, inter- and transdisciplinary analyses. Despite this, and as noted above, speciality fields and academic disciplines do not always coalesce well, limiting the potential for conceptual and disciplinary integration in M&E. Waage et al. observe the lack of effective cross-sector working in complex yet conceptually narrow monitoring practices (4).

Many authors have commented on fragmented and inconsistent M&E in the MDGs. The combination of rates and absolute numbers, the inclusion of indicators that were difficult to define and measure, the addition of indicators during the process, the absence of targets and indicators for the global governance goal, the lack of attention to qualitative elements, and the value of country-level monitoring are examples of critical reflections (9–11). Commenting on the latter, Kenny and Sumner reflect on the on-track/off-track practice of country-level monitoring: ‘the targets associated with particular goal areas do become significantly more ambitious if universally applied at country level, which has become the norm in reports on MDG progress from the World Bank and UN’ (12 p. 10). The flaws in monitoring are reflected in a further disconcerting observation made by these authors: ‘we will not know until at least 2017–2019 which goals were met – and given the lack of baseline data we may never know for some’ (12 p. 9).

Also in terms of concepts and analysis, Waage et al. identify failures to address equity as the ‘most serious shortcoming’ of the MDGs (4 p. 1005). The use of aggregate national statistics to monitor progress, for example, can obscure more localised situations of disadvantage and exclusion. Inadequate analysis of equity can also have broader consequences: ‘the MDGs promote an approach that might systematically exclude individuals at highest risk, achieving improvement on indicators by focusing on those populations that are the easiest to reach’ (4 p. 1007), a phenomenon reflected in a recent analysis of disproportionate aid flows to smaller countries where costs of demonstrating progress were lower (12). In this sense, inadequate analysis of inequity may indirectly contribute to its maintenance.

Finally, the Waage paper observes a pervasive lack of ownership at country level. The goals, targets, and indicators were developed in the global North in a paternalistic process that lacked equivalent attention to the settings and situations into which interventions were applied. As a result (and compounded by the fragmented operations and incomplete metrics described above), the goals, associated projects, programmes, and M&E often lacked relevance and meaning in the contexts where they were implemented (4).

Considering these issues, the following discussion priorities are identified. The first relates to ensuring sufficient political commitment for global health and development post-2015. In light of recent failures to achieve high-level consensus on global climate change and international trade agreements (13–15), it is critical to consider how sufficient political consensus and commitment for global development can be achieved. Second, more effective cross-sectoral working must be prioritised, particularly with regard to ensuring the integrity of state systems. Third, more relevant, inclusive, interdisciplinary analytical frameworks are required to improve notions of what goals are and what success looks like. Responding to the issues surrounding equity, context-specificity, and country-level ownership, improved concepts and analysis of equity and more democratic strategic and operational country-level programming can be viewed as overarching priorities that reconcile the recommendations more generally. In the following section, these themes are considered in light of the current development debate to locate lessons from the past within the current narrative.

## Going forward? The post-2015 sustainable development debate

In June 2012, the Rio+20 United Nations Conference on Sustainable Development was held to reaffirm commitments, review progress, determine how the MDGs will conclude, and to outline a replacement system. The Rio+20 ‘zero-draft’ outcome document *The Future We Want* set out a ‘triple helix’ sustainable development framework consisting of economic development, environmental sustainability and social inclusion (16). *The Future We Want* also identifies six discussion priorities for the agenda setting process: 1) development of a common vision, 2) sufficient political commitment, 3) promotion of a ‘green economy’, 4) enabling institutional frameworks, 5) follow-up frameworks, and 6) means of implementation. The document provides a basis for the development of Sustainable Development Goals (SDGs) that will replace and/or encompass the existing MDGs.

Many analysts were unsatisfied with the conference outputs. Critics noted the lack of high-level political participation, vague, weak texts, and the lack of a sense of urgency to act (17, 18). There were also reactions to the commodification of public goods interpreted in the green economy element (19, 20). Further criticisms related to the lack of reference to health (6, 21, 22): ‘the Rio+20 Summit in June 2012 already raise a red flag for the meagre and weak content of health issues’ (22 p. 33). In response, *The Lancet* joined with the University of Oslo to create a Commission on Global Governance for Health stating that: ‘the Rio+20 conference must not only re-examine, but also put at its centre, the link between health, the environment, and sustainable development’ (21 p. 2217).

**Fig. 2:**
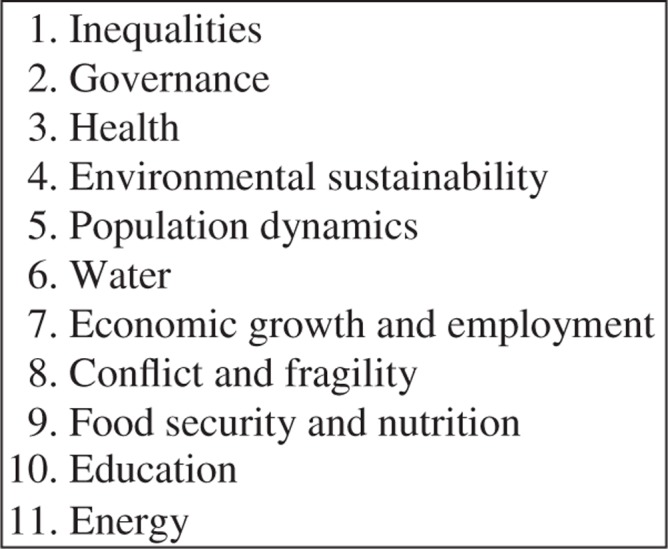
Thematic consultations: High-Level Panel of Eminent Persons on the Post-2015 Development Agenda (25).

Following Rio+20, the UN Secretary General convened the High-level Panel of Eminent Persons on the Post-2015 Development Agenda (HLP). The HLP is co-chaired by President Susilo Bambang Yudhoyono of Indonesia, President Ellen Johnson Sirleaf of Liberia, and Prime Minister David Cameron of the United Kingdom with representatives from government, civil society, academia, youth, and the private sector (23, 24). The HLP is currently engaged in a series of national and thematic consultations, of which one is dedicated to health, sought in part via the online interface www.worldwewant2015.org (Fig. 2) (25). The health consultation is led by an interagency group, UNICEF, WHO, and the Governments of Sweden and Botswana with input from member states, civil society, the private sector, and academia. The consultations will furnish a synthesis report to be published in February 2013, and a dialogue in Botswana in March at which recommendations on health will be made. The HLP presents its overall recommendations to the Secretary General in May prior to the next MDG Summit in September.

A Special Adviser on Post-2015 Development Planning, Amina Mohammed and the UN System Task Team (UNTT) on the Post-2015 UN Development Agenda have also been appointed. Following Rio+20, the UNTT published a roadmap document (9) and a series of background papers reflecting on the MDGs and considering ways forward post-2015, of which one addresses health (26). This paper calls for a new summative health framework rather than an extension of the current condition-specific system, avoidance of rejection of the current goals during this process, and highlights equity, social justice and the social determinants of health as important paradigms for the development of health goal(s). The report is also candid in its recognition of how global goals become interventions in themselves, influencing the content of projects and programmes. Based on their analysis, the UNTT recommends a single high-level health goal post-2015, within which extensions to the MDGs and new priorities can be situated.

**Fig. 3:**
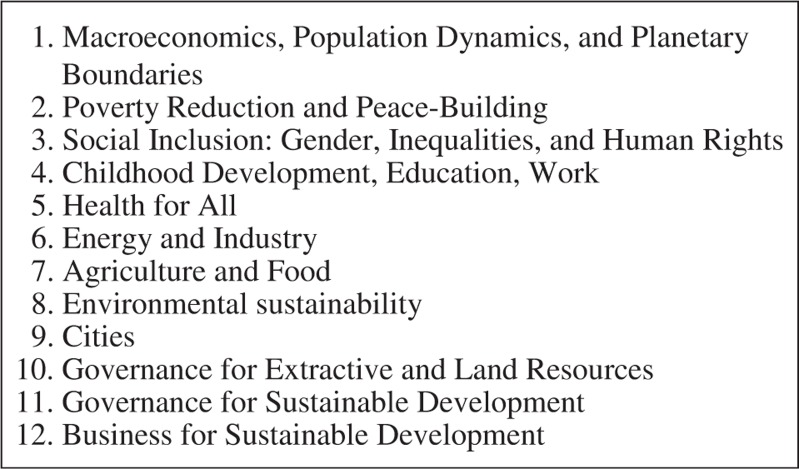
Thematic consultations: Sustainable Development Solutions Network (abridged, 27).

In a parallel stream, the Sustainable Development Solutions Network (SDSN) chaired by Jeffrey Sachs is a group tasked with translating the economic/environmental/social triple helix into SDGs (27, 28). This group also aims to develop novel approaches to sustainable development and to complement the work of the HLP with technical support. The SDSN recently launched the *Solutions Initiative*, introducing programmes to train one million community health workers in Sub-Saharan Africa, and a joint venture with the Italian oil and gas corporation Eni to address access to energy in the region. The group is also conducting thematic consultations with expert groups according to 12 areas that are broadly similar to those of the HLP (Fig. 3). Health is included in this list, articulated in terms of ‘health for all’.

The UN states that ‘these multiple processes aim to ensure that the development framework post-2015 will be borne out of outreach, discussions and consultations geared at capturing the voices, ideas and suggestions of ordinary citizens, different interest and marginalized groups’ (29). The approach is markedly participatory, non-prescriptive and embraces social media in a broadly constructed narrative. It is also appears that the initial omission health has been acknowledged, although in contrast to the MDGs (where health was conceptualised as central to development and directly articulated in three of the eight goals), post-2015, a single goal for health is likely, and likely to be framed in terms of universal health coverage (UHC).

Defined as all people who need care receiving it without financial hardship (30), a resolution on UHC was passed by the UN General Assembly in December 2012 (31, 32). Framed in terms of access to care as part of a health systems approach, UHC consists of access to necessary curative and preventative services that are of sufficient quality, combined with access to financial risk protection (33–35). Speaking at the launch on the SDSN, WHO Director-General Margaret Chan stated that UHC provides a mechanism whereby global health goals can be translated into context and country-specific priorities (36). Several authors have described UHC in terms of a diversity approach whereby: ‘every country will develop its own path, reflecting its own culture and legacy from existing health systems’ (37) p. 861, (38).

Others have observed a tendency for UHC to focus on health financing (39), that positioning health in terms of healthcare ‘misses the point that health is an outcome of policies in many other sectors’ (26 p14), and that consideration of UHC in terms of structural inequities provides a beneficial perspective (40). These observations have led to suggestions that *universal access* (40), and *universal health* (41) may represent more encompassing goals. Otherwise, how vulnerability applies to health systems (as well as to individuals) has been noted, with calls for a ‘voice to the people’ to enhance accountability as part of a democratic approach to health care and health systems (39 p. 2). Further research and debate on how UHC translates into ‘mass free access’ (42 p. 946) to global public goods in an age of austerity is now required to develop the concept and associated metrics.

Elsewhere, calls are being made for renewed systems of global governance. Scholars recommend that following its consultations that the UN convenes a ‘single and networked process for the formulation of goals and of implementations strategies’ (43). Mackey and Liang state the situation in more pressing terms in a recent commentary on the role of WHO: ‘fragmentation due to proliferation of global health actors coupled with inconsistency of financing has created serious challenges’ (44 p. 12). In addition, several authors have reflected on the relevance of traditional aid models as countries transition from low to middle income status (45). Given the consequences of fragmentation observed above, how global governance is organised and related to participatory consultations, whether and how goals should apply to all countries rather than only developing states, and implications for the willingness of high-income countries to commit are further issues for consideration.

Based on the formative horizon, it is reasonable to assert that the on-going discussions focus on how to achieve binding, global political commitment and coherent governance. Attention to how environmental sustainability relates to economic development, and the specifics of where and how health is conceptualised and operationalised are further key considerations. In terms of the latter, detail on the process by which a single health goal is constructed and translated into activity at country level is also required. Finally, it is noteworthy that, despite its presence in the core values,^1^ equity (as distinct from equality) does not feature prominently in the emerging post-2015 and SDG consultative frameworks. Given the discussion priorities identified above, it is asserted that equity should be prioritised in more comprehensive and binding terms post-2015. This assertion is developed below.

## Centralising equity

From an analytical perspective, it is perhaps most troubling that incomplete analysis of equity can inadvertently maintain disadvantage and exclusion. Based on this observation and the recommendations set out above, in this section equity is considered in terms of concepts and analysis, and in terms of programming and operations.

### Equity in analysis

To overcome the limitations of health equity metrics, scholars have urged attention to *mechanisms* of distribution as well as distribution *per se* (46–48). This implies attention to why resources become unequally and unfairly distributed, and from a public health perspective, how this manifests in avoidable illness and disease. Distribution is a core element of the sustainable development framework presented in the report *Transforming Innovation for Sustainability* published by the ESRC STEPS Centre (Social, Technological and Environmental Pathways to Sustainability) in 2012 (49). Here, the authors call for attention to how development assistance occurs, by whom and for what purpose. The group acknowledge that these issues ‘ultimately reflect political values’ that bring ‘further and ultimately more fundamental socio-political and justice questions to bear’ (49 p. 8). Waage et al. also make reference to political influences on distribution in a cautionary comment on post-2015 development: ‘aspirations for human development co-exist in a globalised political economy that is marked by substantial inequality and exploitation’ (4 p. 1015). If global political and economic forces influence mechanisms of distribution, which in turn influence health, then it follows that analysis of health equity must encompass the political context of its production.

Despite its relevance, the analysis of politics in public health is rare. Vicente Navarro has repeatedly identified the absence of the ‘political determinants of health’ from normative analytical paradigms, asserting that because most research sponsors are public bodies with implicit or explicit political agendas, serious conflicts of interest may exist regarding what types of research to support, and which types of factors to intervene upon (50, 51). Public health research, in turn, tends to avoid political issues, aligning with more neutral agendas, that attract funding and/or other forms of support, but that risk neglecting the root causes of avoidable and unfair burdens of illness and disease.

Examining the structural contexts of health inequities also implies a focus on the influence of the private sector in public affairs. With unprecedented growth in recent decades, multinational corporations have strong political interests related to market freedom and the pursuit of profit for growth that are pursued and enacted through aggressive lobbying and legislative influence. Despite its emphasis on individual freedoms, neoliberal structural drivers have been observed to erode self-determination particularly among disadvantaged and vulnerable groups, reduce social cohesion, and result in poorer health outcomes (52). Given their presence in development architecture (poverty reduction strategies, social welfare arrangements, and the MDG framework [4, 53, 54]), how neoliberal ideologies underpin public policy and implications for the provision of public goods also warrant attention.

### Equity in operations

If the analysis of equity implies the analysis of politics, and if the sensitivity of politics precludes it from analysis, then a useful function of the post-2015 development debate may be enabling states to critically address matters of development, distribution and the structural and political determinants of health inequities. Researchers at the STEPS Centre call for a system of global governance that prioritises linkages between science and society through citizen-led movements and locally defined agendas, moving away from elite science towards local organising to develop technologies relevant to particular contexts (55, 56). In the report *Transforming Innovation for Sustainability*, the authors identify the need for bottom-up streams of innovation, calling for a ‘far greater recognition and power to grassroots actors and processes, involving them within an inclusive, multi-scale innovation politics’ (49 p. 1). The STEPS advocate for ‘distributed development’ as critical for the ‘democratic legitimacy of any post-2015 framework’ in recent written evidence to the UK parliamentary committee on international development. Echoing Margaret Chan, the group urge global monitoring centred around local adaptation and suggest capacity building and process-based evaluation that represents the perspectives of societal groups (43).

Equity is also a matter of fairness, justice, and human rights. Despite the centrality of rights to health and development (57–62), scholars have observed how principles of equity and social justice were lost in translation as the Millennium Declaration was operationalised in goals, targets and indicators (63–65).^2^ In response, and based on a global tobacco control treaty (66), the Joint Action and Learning Initiative on National and Global Responsibilities for Health (JALI) at the O'Neill Institute for National and Global Health Law at Georgetown University has produced a Framework Convention on Global Health (FCGH) that develops responsibility for global health according to a human rights paradigm. The FCGH seeks to formalise responsibilities for health equity through legal infrastructure based on an observed fundamental conflict in normative aid models: ‘health is a globally shared responsibility, reflecting mutual risks and vulnerabilities – an obligation of health justice that demands a fair allocation of burdens and benefits. International funding should be seen as a partnership designed to achieve the communal objective of safeguarding health and narrowing inequalities’ (8 p. 2089). JALI seeks to develop the FCGH through engagement with a range of stakeholders, creating a political space for mutual and distributed accountability codified in, and enabled through, binding legal commitments (67, 68). The redistributive approach was reflected in JALI's contribution to an HLP consultation in November, where calls were made for the post-2015 discussion to: ‘be slowed to enable meaningful input from communities that cannot participate in online consultations and presently lack the means to participate in – much less have information about – other consultative processes’ (69 p. 13).

STEPS and JALI represent complementary streams of debate that connect civil society and social activism to state structures and global governance as part of a redistributive social justice approach. Both acknowledge that their agendas imply radical change, and indeed call for it. These and other critical perspectives (70–74) should be given due exposure as the debate continues to confront fundamental and ideological conflicts between concepts such as economic growth and human development as a capabilities approach.

## Conclusions

The global landscape is changing. Virtually all reporters acknowledge a global context indistinguishable from the one in which the MDGs were formulated. Climate change, the environment, planetary boundaries, globalisation, the global economic crises, urbanisation, industrialisation, migration, the double burden of non-communicable and infectious diseases, conflict, violence, fragility, and ageing populations are a handful of the ever-more globalised issues that programming and monitoring must be capable of responding to. As stated by the UNTT: ‘It is no longer viable to think of solutions in terms of individual sectors’ (26 p. 10).

In addition, and despite the significant achievements of the MDGs, 1.4 billion remain in conditions of extreme poverty, over 900 million are affected by chronic hunger, universal primary completion and mortality goals are unlikely to be met, and the progress that has been achieved is markedly uneven within and between countries (9–12): ‘part of the explanation for this stagnation in progress lies in a failure to reach the most vulnerable populations … these gaps within and between countries demand a much sharper focus on inequities and their consequences for health’ (26 pp. 4–5). Rooted in the progressive realisation of rights *and* economic development, UHC is a promising concept. Recent evidence is suggestive of substantial gains, although authors point to the need to consider UHC as a diversity approach that addresses structural inequities and democratic accountability (75, 76).

This review concludes that prioritising *universal health equity* post-2015 may offer instrumental and substantive co-benefits that subscribe to the original terms of the Millennium Declaration, that respond to the limitations observed in current M&E practice, and that enable attention to the root causes of intractable health problems. As the circularity between measurement and policy becomes more explicitly acknowledged, it is clear that these topics must be measured if they are to be addressed. For more authentic renditions of how and why inequities occur and recur in particular settings and for particular groups, analyses that attend to the structural, political, and economic contexts of health production and distribution, to the globalised political ideologies that underpin public policy, and to the manifest implications for population health are required in broader and more integrated analytical frameworks than exist in the mainstream at present.

Finally, because analysis of equity gives rise to issues traditionally beyond the scope of public health research, a shift in the normative analytical paradigm is also necessary. In this sense, 2015 can be viewed as an opportunity to define a new global health orthodoxy that enables more democratic and critical perspectives through reform of global governance and by fostering inclusion and participation. Based on these observations, discussions on critical and collective cross-sector, country-led dialogues as part of an approach characterised by investment at the national level ensuring continuity, predictability and coherence is recommended to attend to the structural contexts of health inequities, to examine mechanisms by which health and risk are conditioned and produced, and to develop regulatory frameworks to protect fundamental rights and freedoms so that those for whom health and development issues are most relevant define the way forward post-2015.

## Footnotes

1 In the report *Realizing the Future We Want For All*, the UNTT set out a vision for the future of development comprising three core values: human rights, equality and sustainability. From this, four key dimensions are derived: inclusive social development, environmental sustainability, inclusive economic development and peace and security (10).

2 The core values of the Millennium Declaration are freedom, equality, solidarity, tolerance, respect for nature and shared responsibility (56).
